# Capacitance-Based Untethered Fatigue Driving Recognition Under Various Light Conditions

**DOI:** 10.3390/s24237633

**Published:** 2024-11-29

**Authors:** Cheng Zeng, Haipeng Wang

**Affiliations:** 1 School of Advanced Manufacturing, Nanchang University, Nanchang 330031, China; 2 School of Intelligent Manufacturing, Jiangsu College of Engineering and Technology, Nantong 226006, China

**Keywords:** capacitance, blink detection, fatigue driving recognition, neural network

## Abstract

This study proposes a capacitance-based fatigue driving recognition method. The proposed method encompasses four principal phases: signal acquisition, pre-processing, blink detection, and fatigue driving recognition. A measurement circuit based on the FDC2214 is designed for the purpose of signal acquisition. The acquired signal is initially subjected to pre-processing, whereby noise waves are filtered out. Subsequently, the blink detection algorithm is employed to recognize the characteristics of human blinks. The characteristics of human blink include eye closing time, eye opening time, and idle time. Lastly, the BP neural network is employed to calculate the fatigue driving scale in the fatigue driving recognition stage. Experiments under various working and light conditions are conducted to verify the effectiveness of the proposed method. The results show that high fatigue driving recognition accuracy (92%) can be obtained by the proposed method under various light conditions.

## 1. Introduction

Fatigue can be described as a mental state that results from a reduction in alertness between wakefulness and sleepiness. When drivers are fatigued, they are more susceptible to experiencing problems such as distraction, a slow response time, and operational errors. Driving safety can be enhanced by the early detection of fatigue and timely intervention of fatigue driving [1,2,3].

In driving fatigue, there are observable changes in physiological indicators or physiological activities (e.g., brain activity, heart rate, blinking) that can be measured and quantified. Therefore, the presence of driving fatigue can be detected by the monitoring of human physiological indicators.

The Electroencephalogram (EEG) signal is an important indicator of brain activity, with a particular correlation to driving fatigue [4,5]. The state of driving fatigue can be identified by analyzing the EEG signals obtained from electrodes that are fixed on the driver’s head [6]. Furthermore, several typical characteristics of the Electrocardiogram (ECG) signal, including heart rate variability [7], low-frequency energy [8], and high-frequency energy [9], can also be employed as a basis for fatigue judgement. Typical radars, including the UWB radar [10,11], Doppler radar [12], and mm-wave radar [13,14], can all be utilized for the detection of blinks based on the Doppler effect. Dong et al. [15] made use of mm-wave radar to obtain the driver’s heart rate and to identify the driver’s state of fatigue. Fan et al. [16] evaluated fatigue by quantifying the amplitude and mean frequency of the driver’s Electromyogram (EMG) signal. The peak power spectrum and center frequency of the pulse signal are found to be closely related to fatigue. Li et al. [17] employed a methodology for fatigue detection based on the analysis of pulse signals. When the driver is fatigued, there is a discernible change in the blink rate and blink time [18,19]. The construction of a fatigue driving detection model combined with a genetic algorithm and a generalized regression neural network is based on the mean value, standard deviation, and root-mean-square characteristics of the eye electrical signal [20].

The aforementioned methods are effective in accurately detecting fatigue; however, they require direct physical contact with the human body, which is a more invasive and inconvenient procedure for the driver. Conversely, visual image analysis-based blink detection methods have attracted much attention in recent studies. The assessment of the driver’s fatigue status can be more effectively determined by analyzing their facial features.

Due to their non-contact characteristic, RGB image analysis-based blink detection methods have attracted a lot of attention [21,22]. Knapik et al. [23] proposed a fatigue driving detection method based on thermal imaging yawning detection, which detects human fatigue by detecting changes in oral temperature. Kiashari et al. [24] proposed a fatigue detection system based on thermal imaging to analyze drivers’ breathing characteristics. The classification model is trained by calculating respiration rate and inspiratory expiratory time ratio. de Lima Medeiros et al. [25] proposed a convolutional neural network (CNN) and a Support Vector Machine (SVM) for blink detection. Jordan et al. [26] proposed a CNN-based blink detection method for estimating the driver’s drowsiness level. Ryan et al. [27] designed a fully convolutional recurrent neural network to detect and track facial features for blink pattern analysis based on an event camera.

However, the aforementioned methods based on visual image analysis have privacy issues and are also sensitive to light conditions. Compared with the above methods, measurements based on capacitance [28,29] have received much attention recently due to their non-contact and real-time methodology, and applicability in complex environments. The methods [28,29] focus on facial and head gestures, which are achieved by detecting changes in capacitance during 12 different facial expressions and head gestures. Liu et al. [30] proposed a non-contact and real-time blink detection method based on capacitive sensing, which can detect blinks in complex environmental conditions. Compared with Liu et al.’s method, both methods have blink detection, but our method adds a band-pass filtering step, which can better extract specific data on eye closing time and eye opening time. Moreover, Liu et al.’s method only focuses on blink detection, while our method further investigates the relationship between blinking and driving fatigue. Also, we use two electrodes placed on two lenses, respectively, Liu et al.’s method only uses one electrode placed on a single lens. This enables us to effectively carry out subsequent fatigue driving recognition.

To address the above problems, this study proposes a modified capacitance-based eye-blink detection method for fatigue driving under various light conditions. In this method, an electrode made of copper foil is glued to the frame of the eyeglass. The electrode is connected to a FDC2214 chip-based capacitance measurement circuit. An STM32 board is connected to the FDC2214 chip, which conducts all the data processing tasks. The data processing includes three main steps: pre-processing, blink detection, and fatigue driving recognition. Experiments are carried out under various working and environmental conditions to verify the effectiveness of the proposed method.

The main contributions in this paper are summarized as follows:

- This paper designs a reading circuit for parasitic capacitance that can implement the reading of parasitic capacitance with the person’s eyes open and closed, and measure the blink time.
- This paper proposes a capacitance-based fatigue driving recognition approach that is non-contact, light-insensitive, and applicable in complex environments. This method classifies individuals’ fatigue scale and constructs a neural network to assess individuals’ fatigue levels.
- This paper performs accuracy analysis across various subject statuses and various light conditions to demonstrate that the proposed method is not affected by complex environments, further validating the robustness of the method.

The remainder of this paper is organized as follows. Section 2 details the fatigue driving recognition algorithm procedure. Section 3 shows the original signal acquisition and analysis. Section 4 details the data pre-processing algorithm. Section 5 shows the detailed blink detection algorithm. Section 6 shows the fatigue driving recognition algorithm. Section 7 analyzes the experimental results and Section 8 concludes the paper.

## 2. Details of the Fatigue Driving Detection Algorithms

**Figure 1:**
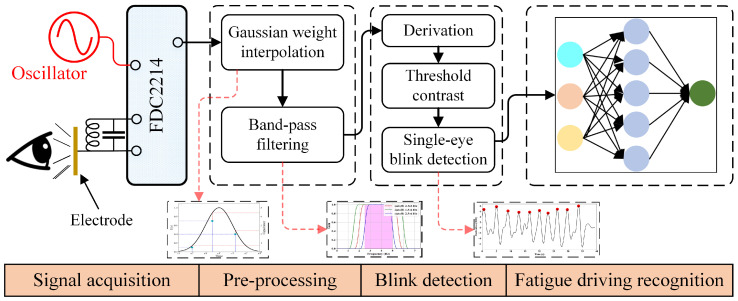
Details of the fatigue driving recognition procedure.

Figure 1 details the fatigue driving recognition procedure. The electrode is placed in front of the human eye, and connected to a measurement circuit based on the FDC2214 chip. The measurement circuit measures the real-time capacitance signal on the electrode. The measured data are then processed in three steps:

**Pre-processing:** the ambient noise wave is first filtered out by Gaussian weight filtering. A band-pass filter is then used to filter out clutter outside the human blink frequency range.

**Blink detection:** differential processing is first performed on the filtered data, and then the differential value is compared with the threshold value frame by frame. The blink characteristic (the eye closing time, eye opening time, and idle time) is identified according to the comparison result.

**Fatigue driving recognition:** the blink characteristic is analyzed by a neural network. The neural network input includes the eye closing time, eye opening time, and idle time. The neural network outputs the driving fatigue scale.

## 3. Signal Acquisition and Analysis

**Figure 2:**
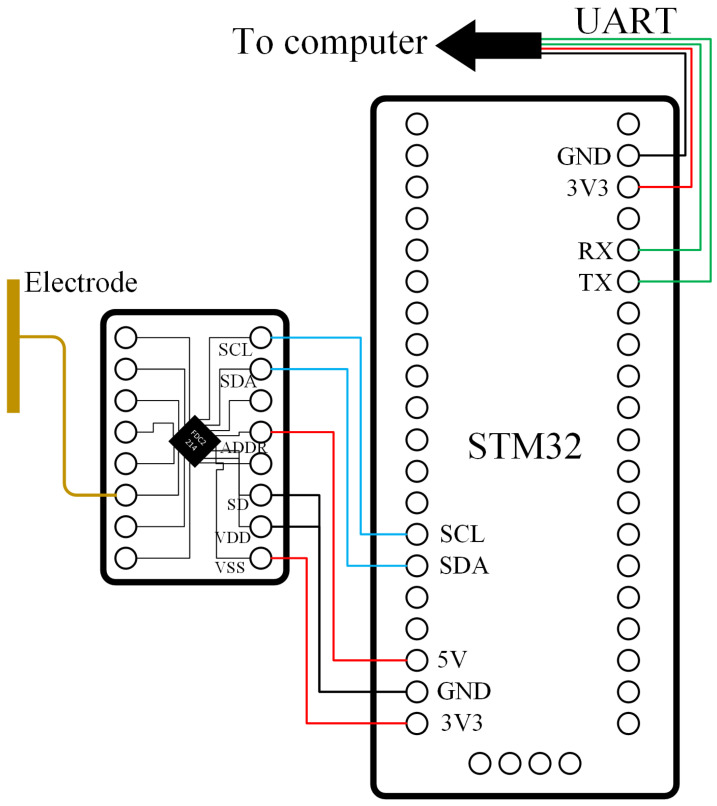
Signal acquisition and analysis circuit.

Figure 2 shows the signal acquisition and analysis circuit. The circuit consists of an electrode, a measurement circuit based on the FDC2214 chip developed by Texas Instruments (Dallas, TX, USA), and an STM32 board developed by STMicroelectronics. The electrode is connected to the measurement circuit by copper wire. The measurement circuit is connected to the STM32 board. The STM32 board supplies power to the measurement circuit, and communicates with the measurement circuit using the IIC communication protocol. The signal analysis is conducted on the STM32 board.

The measurement circuit is designed based on the RLC parallel resonance principle. The electrode is equivalent in parallel with an inductor, L, and a capacitor, C. The other side of the electrode is equivalent to the ground. The resonance frequency can be obtained from the following equation:

(1) $$f_{x}=\frac{1}{2\pi \sqrt{L(C+C_{x})}}$$

where *L*, *C*, and $C_{x}$ represent the inductor impedance, the capacitor capacitance, and the equivalent capacitance value of the human eye, respectively. In this paper, *L* and *C* are constants and the equivalent capacitance resistance of the human eye is related to the following factors:

(2) $$C_{x}=\frac{\epsilon S}{4\pi kd}$$

where ϵ represents the equivalent dielectric constant, *S* and *k* represent the plate area and electrostatic constant, respectively, and *d* is equivalent to the distance between two plates.

**Figure 3:**
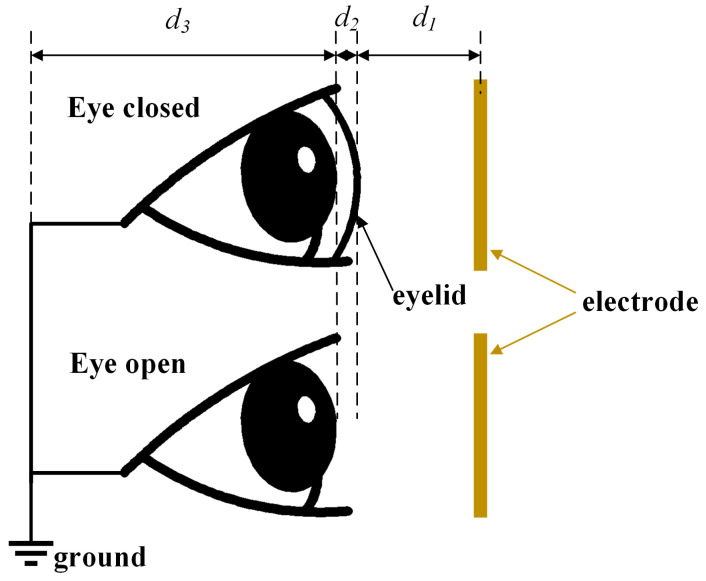
Overview of the equivalent capacitor.

Figure 3 shows the overview of the equivalent capacitor. The electrode (one plate) is located in front of the human eye at a fixed distance. The other plate is equivalent to the ground. When the person closes their eyes, the objects between the two plates can be equivalently divided into three modules: the air between the electrode foil and the eyelid, the eyelid, and the other parts. There is:

(3) $$\frac{1}{C_{x}}=\frac{1}{C_{1}}+\frac{1}{C_{2}}+\frac{1}{C_{3}}$$

and:

(4) $$C_{i}=\frac{\epsilon_{i}S}{4\pi kd_{i}},i\in \left\{1,2,3\right\}$$

where $\epsilon_{1}$, $\epsilon_{2}$, and $\epsilon_{3}$ represent the equivalent dielectric constants of the air, the eyelid and other parts, respectively. $d_{1}$, $d_{2}$, and $d_{3}$ represent the equivalent thickness of the air between the electrode and the eyelid, the thickness of the eyelid, and the equivalent thickness of other parts, respectively. There is:

(5) $$C_{x}=\frac{S}{4\pi k(\frac{d_{1}}{\epsilon_{1}}+\frac{d_{2}}{\epsilon_{2}}+\frac{d_{3}}{\epsilon_{3}})}$$

when the person opens their eyes, the eyelids do not appear in front of the electrode, there is:

(6) $$\frac{1}{C_{x}^{\prime }}=\frac{1}{C_{12}^{\prime }}+\frac{1}{C_{3}^{\prime }}$$

where:

(7) $$\left\{\begin{array}{c} C_{12}^{\prime }=\frac{\epsilon_{i}S}{4\pi k(d_{1}+d_{2})}\\ C_{3}^{\prime }=\frac{\epsilon_{3}S}{4\pi kd_{3}} \end{array}\right.$$

Combining the above formula, there is:

(8) $$C_{x}^{\prime }=\frac{S}{4\pi k(\frac{d_{1}+d_{2}}{\epsilon_{1}}+\frac{d_{3}}{\epsilon_{3}})}$$

(9) $$\frac{C_{x}}{C_{x}^{\prime }}=\frac{\frac{d_{1}}{\epsilon_{1}}+\frac{d_{2}}{\epsilon_{1}}+\frac{d_{3}}{\epsilon_{3}}}{\frac{d_{1}}{\epsilon_{1}}+\frac{d_{2}}{\epsilon_{2}}+\frac{d_{3}}{\epsilon_{3}}}$$

The dielectric constants of the air and water are 1 and 100, respectively. Since most of the human body is water, the dielectric constant of the eyelid is close to 100. The dielectric constant between the plates varies when the eyes are open and closed. Therefore, the measurement value will mutate when the person blinks. The human blink activity can be detected by analyzing the mutation.

## 4. Data Pre-Processing

### 4.1. Gaussian Weight Interpolation

The STM32 board conducts the high-frequency signal acquisition and all the data processing in this study. In data pre-processing, the original data are first interpolated for a fixed period. Assuming that the data collection period is *T*, the interpolation in the *n*th period is the weighted sum of the values near it. The weights are designed as follows:

(10) $$f\left(t\right)=\frac{1}{\sqrt{2\pi }}e^{-\frac{{\left(t-nT\right)}^{2}}{2}}$$

where f(t) represents the weight value at time *t*. The fitted data at time nT is:

(11) $$F^{\prime }\left(nT\right)=\sum_{t\in t^{*}}f\left(t\right)F\left(T\right)$$

where $F^{\prime }\left(nT\right)$ represents the fitted value at time nT. F(t) represents the measured value at time *t*, and $t^{*}$ represents the set of all measured time near time nT.

### 4.2. Band-Pass Filtering

Generally, human blink time ranges from 0.2 s (5 Hz) to 0.4 s (2.5 Hz). Due to the problems of sensor error and environmental noise, the initially acquired data have many clutter waves outside the human blink frequency. In this section, a Butterworth band-pass filter is used to filter out the high-frequency and low-frequency clutter waves.

The magnitude of the first-order frequency response is:

(12) $$H\left(j\omega \right)=\frac{j\omega \tau_{1}}{1+j\omega \tau_{1}}\frac{1}{j\omega \tau_{2}}$$

(13) $$\left[W_{1},W_{2}\right]=\left[\frac{1}{\tau_{1}},\frac{1}{\tau_{2}}\right]$$

where $\left[W_{1},W_{2}\right]$ represents the passband. As can be seen from the equations, the filter performance is affected by two factors: the filter order and the filter passband.

**Figure 4:**
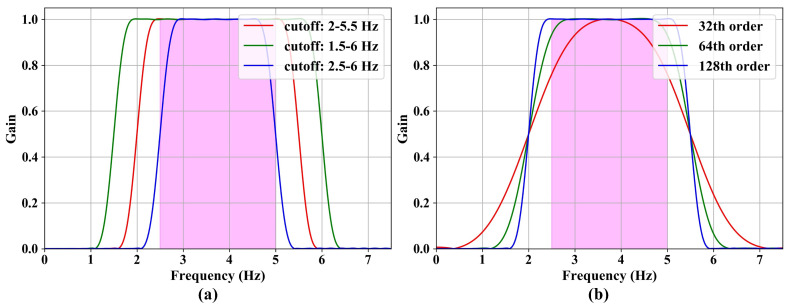
The frequency response of the band-pass filter: (**a**) the frequency response of a 128th order filter at various cutoffs; (**b**) the frequency response of a [2.0, 5.5] cutoff at various filter orders.

Figure 4 shows the frequency response of the band-pass filter under various filter orders and passbands. The pink zone represents the frequency range of the human eye blink. Figure 4a shows the frequency response of a 128th order filter at various cutoffs. Figure 4b shows the frequency response of a [2.0, 5.5] cutoff at various filter orders. It can be seen that the stopband falls faster as the filter order increases, and the stopband widens as the passband widens. Based on the above analysis, a 128th order band-pass filter with a [2.0, 5.5] passband is used.

**Figure 5:**
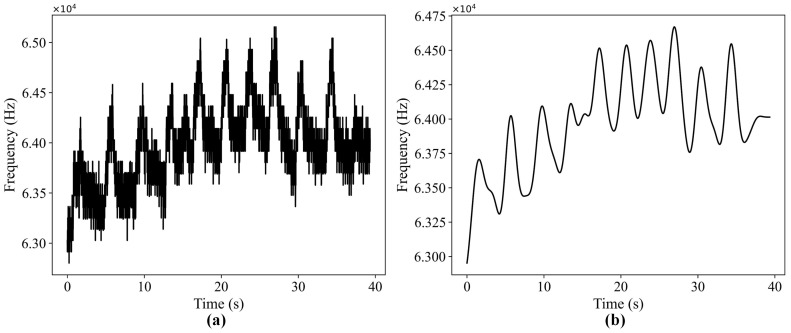
Results of the band-pass filtering: (**a**) the original data; (**b**) the filtered curve.

The subject is asked to blink 10 times in approximately 40 s. The original data are shown in Figure 5a. As can be seen from the figure, a large amount of noise exists in the original data. Blink-caused frequency fluctuation cannot be detected clearly, and therefore cannot be used directly for blink detection. Figure 5b shows the filtered curve. It can be seen from the figure that the noise is effectively removed after the band-pass filtering. The frequency fluctuation caused by the blink is more obvious.

## 5. Blink Detection Algorithm

The blink detection algorithm is shown in Algorithm 1. The input to the algorithm is a series of frequency values measured on human eye in the past N time steps. The algorithm first differentiates the time series data and then compares the differentiated data with the threshold. If the data exceed the threshold, the blinking behavior is considered as a possible occurrence. The confidence value of the blink is related to the value after differentiation and the number of time steps exceeding the threshold. The blink is considered to have occurred when the fused confidence value is greater than 1. 

**Algorithm** **1:** Blink detection

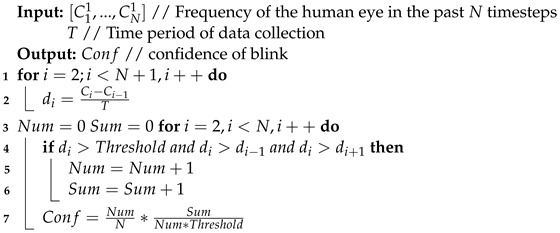



**Figure 6:**
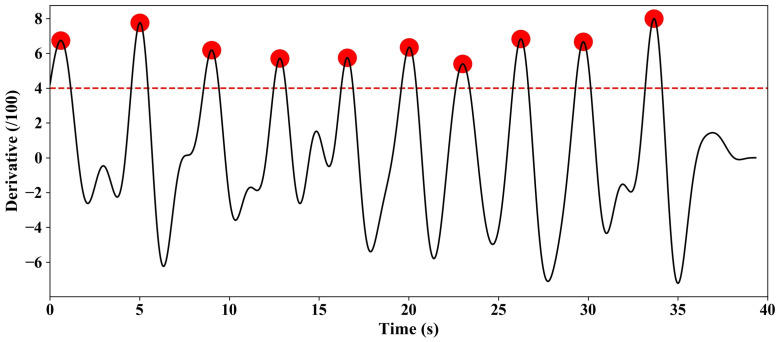
Derivative of frequency with time.

Figure 6 shows the derivative of the frequency with respect to time. The original data are shown in Figure 5b. It can be seen from the figure that the data are more regular after differentiation. In this paper, the threshold is set at 4.0. The person is considered to have blinked once if the peak of the curve after differentiation is greater than the threshold. It can be seen that there are a total of 10 peaks. The time of the 10 peaks corresponds to the human blink times.

## 6. Fatigue Recognition Algorithm

In this section, a neural network for fatigue driving recognition is constructed. For the training of the neural network, the Fatigue Self-assessment Scale (FSAS) is used for human fatigue scale assessment. Subjects are asked to complete an FSAS form, and the blink characteristics (the eye closing time, eye opening time, and idle time) are simultaneously recorded. The neural network establishes the relationship between the blink characteristics and the fatigue scale.

### 6.1. Fatigue Self-Assessment Scale

Table 1 refers to how the subject feels. There are 10 fatigue statements, and each fatigue statement is divided into five categories (i.e., Never, Sometimes, Regularly, Often, Always) based on different fatigue scales. We assign a score of 1 to 5 to the five categories, respectively. If the subject scores 1 point for each category, the total score is 10 points. If the subject scores 5 point for each category, the total score is 50 points. Therefore, each subject’s score varies from 10 (the lowest level of fatigue) to 50 (the highest level of fatigue).

### 6.2. Fatigue Level Computing Neural Networks

**Figure 7:**
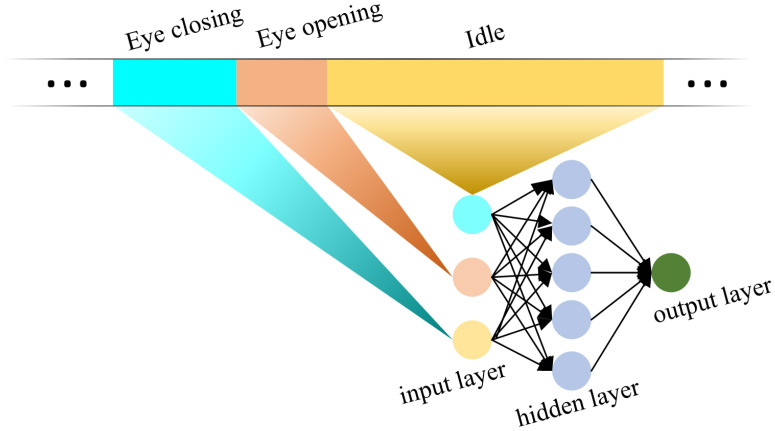
Fatigue level computing neural network.

Figure 7 shows the fatigue scale computing neural network. The neural network has three layers: the input layer, the hidden layer, and the output layer. There are a total of three neurons in the input layer, corresponding to the human eye closing time, eye opening time, and idle time. The hidden layer has five neurons. The output layer has one neuron representing the human fatigue scale.

**Figure 8:**
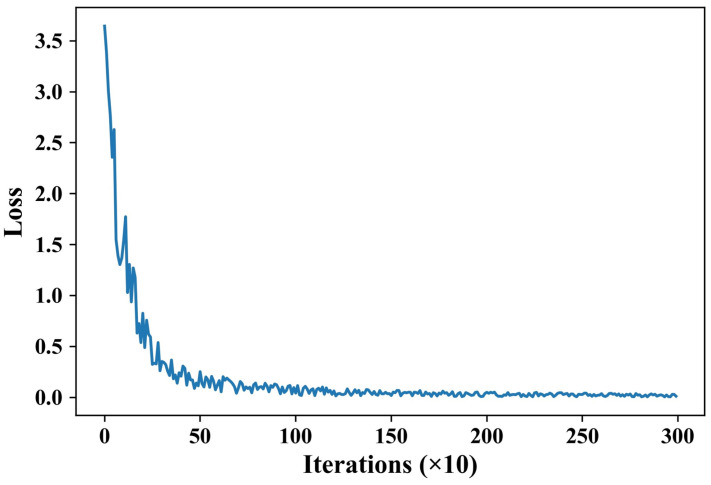
Iteration curve of the neural network.

A total of five subjects participate in the data collection. The data are collected at 7 p.m., 9 p.m., 11 p.m., 1 a.m., and 3 a.m. During the data collection process, the subjects are asked to sit, stand, and walk at a constant speed. Subjects are asked to complete the fatigue self-assessment form immediately after each data collection. The video size of each subject during the experiment is about 260 s. The sliding window size for data extraction is 40 s, and the sliding window step size is 2. Due to the inaccurate video size of 260 s for each subject during the experiment, a total of 8343 data sets are collected, 80% of which are used for training. The iteration curve is shown in Figure 8. As can be seen from the figure, the loss gradually decreases as the number of iterations increases. The iteration curve reaches stability when the number of iterations reaches 1500 times. The remaining 20% is used for testing. The accuracy is calculated using the following formula:

(14) $$r=1-\frac{\sum_{i=1}^{1669}\frac{n_{i}-f_{i}}{f_{i}}}{1669}*100\%$$

where $n_{i}$ represents the *i*th fatigue level computing result, and $f_{i}$ represents the *i*th fatigue level self-assessment. The results show that the proposed algorithm achieves an accuracy rate of 93.6%.

## 7. Experimental Results and Analysis

**Figure 9:**
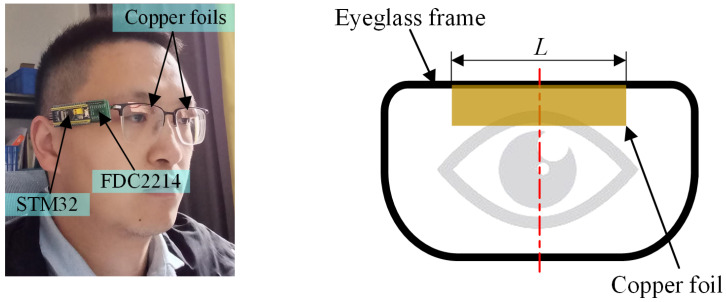
The electrode position on the eyeglass.

**Figure 10:**
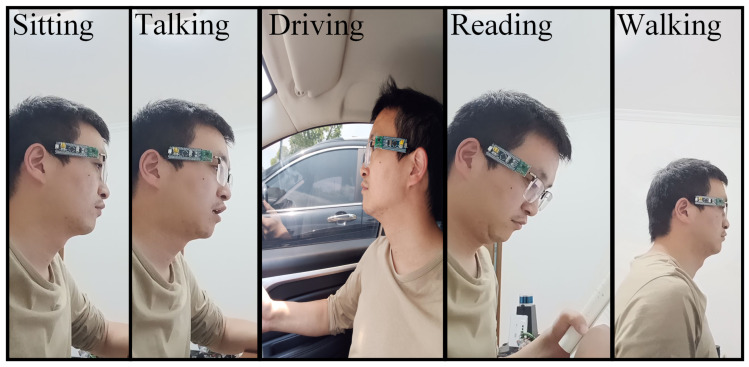
The experimental scenes.

The electrode is positioned above the eyeglass frame (see Figure 9). The width and thickness of the electrode are 8 mm and 0.1 mm, respectively. Figure 10 shows the experimental scenes designed in this paper. The blink detection system, including a bottom plate, an STM32 board, an FDC2214-based measurement circuit, and an electrode, is fixed on the glass leg. The experiments are carried out in various experimental scenarios and light conditions to verify the performance of the proposed method. A total of five subjects participate in the experiment. The follow-up experiments are conducted at different times (7 p.m., 9 p.m., 11 p.m., 1 a.m., and 3 a.m.) under each experimental condition. In each data set, the subject is required to blink 10 times in 40 s.

The fatigue scale is compared under various human statuses (sitting walking, reading, talking, and driving). All the experiments in this group are conducted under natural light conditions.

- **Sitting:** the subjects sit still at a table;
- **Walking:** the subjects walk smoothly;
- **Reading:** the subjects sit still at a table reading a book;
- **Talking:** the subjects sit still at a desk and speak to the computer;
- **Driving:** the subjects drive on safe roads at speeds below 60 km/h.

### 7.1. Analysis of Blink Detection Accuracy Under Various Electrode Lengths

**Figure 11:**
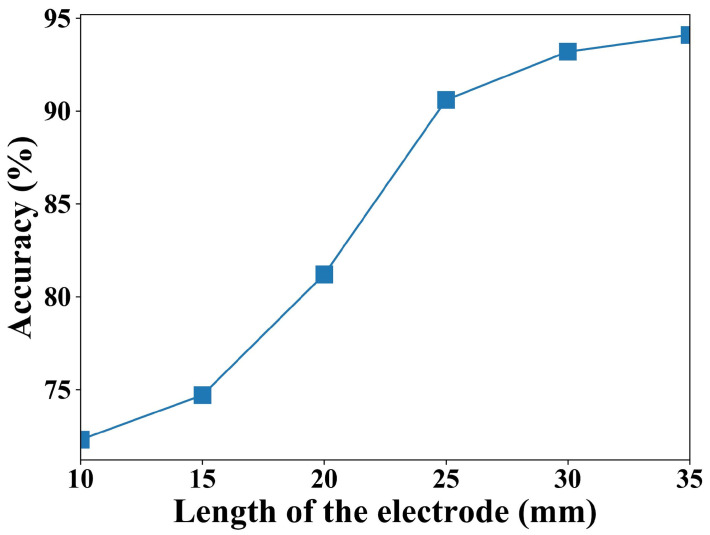
The blink detection accuracy under various electrode lengths.

The accuracy is shown in Figure 11. As can be seen from the figure, the accuracy increases as the electrode length increases. The main reason for this is that the blink-caused capacitance fluctuation is small when the electrode length is short. It is difficult to effectively isolate the capacitance fluctuation in the subsequent signal processing. The experimental results show that an accuracy of more than 90% can be obtained when the electrode length is longer than 25 mm. In the subsequent experiments, the electrode length is set to 25 mm.

### 7.2. Analysis of Measurement Accuracy Under Various Subject Statuses

**Figure 12:**
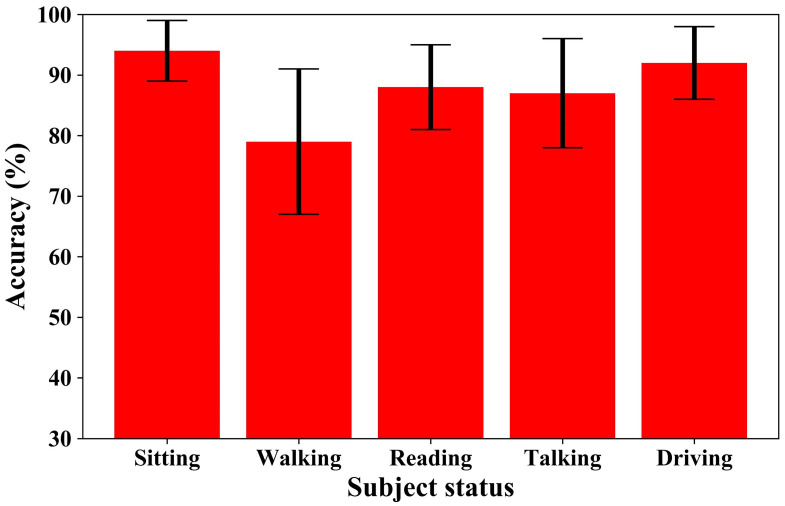
The measurement accuracy under various subject statuses.

To investigate the effectiveness of capacitance-based fatigue recognition, we compare the fatigue measurement accuracy under various subject statuses. The calculation result is shown in Figure 12. As can be seen from the figure, high measurement accuracy can be achieved under various human statuses. Among them, the accuracy is the highest (94%) when the subject is sitting, and the lowest (79%) when the subject is walking. The error band is also the smallest when the subject is sitting, and the largest when the subject is walking. The main reason for this is that the glass shakes inevitably when the subject is walking, which will lead to an increase in noise waves. In the driving status, an accuracy of 91% is achieved.

### 7.3. Analysis of Fatigue Driving Recognition Accuracy Under Various Light Conditions

**Figure 13:**
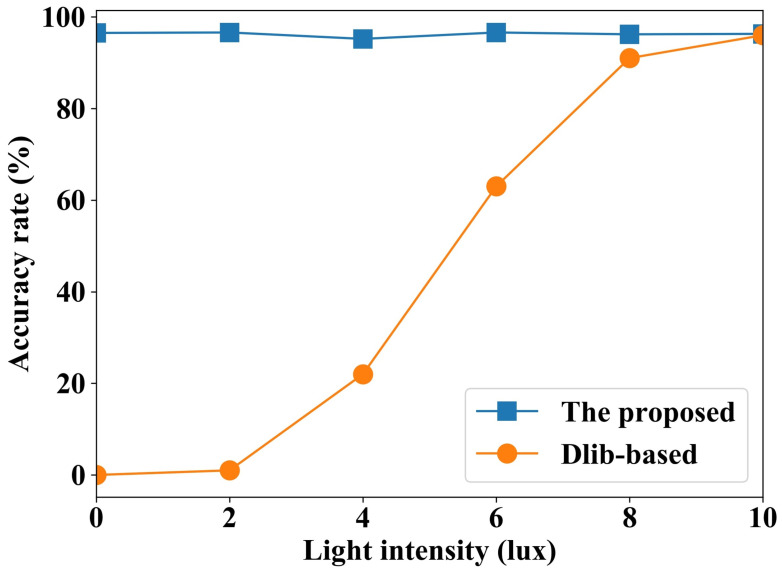
Fatigue driving recognition accuracy under various light intensities.

To investigate the robustness of our proposed method to light in complex environments, we compare the accuracy under various light conditions. In the driving status, the human fatigue scale is studied under various light conditions. The results are compared using the RGB camera-based blink detection algorithm. In the control group, the dlib library [31] is used for human face key points detection. The key points are used to identify human blink characteristics, and then the fatigue level is also calculated by the neural network constructed in this study. The accuracy comparison is shown in Figure 13.

As can be seen from Figure 13, the accuracy of the RGB image-based method improves as the light intensity increases. The performance is the same as with the proposed methods in this study when the light intensity reaches 10 lux. The reason for this is that the method cannot extract effective face key features at low light intensities, and thus cannot effectively recognize fatigue driving. In contrast, the performance of the proposed method is not affected by the light intensity, and high fatigue driving accuracy can be obtained under various light intensities. The main reason is that the capacitance measurement is not affected by the light conditions.

## 8. Conclusions and Future Work

### 8.1. Conclusions

To address the problem of low recognition accuracy of fatigue driving under complex environmental conditions, a capacitance-based fatigue driving recognition method is proposed in this study. The method consists of four key steps: signal acquisition, pre-processing, blink detection, and fatigue driving recognition. The measurement circuit based on the FDC2214 chip is used to obtain the high-frequency capacitance signal in front of the human eye. The acquired signal is filtered for clutter in the pre-processing stage, and then the blink characteristics are accurately identified in the blink detection stage. The human blink characteristics include eye closing time, eye opening time, and idle time. Finally, the BP neural network calculates the fatigue scale in the fatigue recognition stage. The three parameters of eye closing time, eye opening time, and idle time are used as the neural network input. Finally, a series of experiments under various usage and environmental conditions are conducted to verify the effectiveness of the proposed method, and the following conclusions are drawn:

(1) For high fatigue driving recognition accuracy, the electrode length should be longer than 25 mm.

(2) The capacitance-based blink recognition method can perform blink detection under various human statuses (sitting, walking, standing, talking, reading, driving, etc.).

(3) High fatigue driving recognition accuracy (92%) can be obtained by the proposed method under various light conditions.

### 8.2. Future Work

In future work, it is necessary to introduce more objective ground truth (e.g., EEG [6], ECG [7], sEMG [16], SpO2 [32], and reaction time [33]), and utilize technologies such as multi-sensor information fusion to comprehensively measure fatigue levels. And this article only uses the FSAS form to evaluate the fatigue levels, which is a subjective ground truth. In addition, blink detection based on capacitance is also affected by various factors, such as the subject touching the glass frame with their hands, which can cause changes in capacitance and affect the detection results. Hence, more in-depth research will focus on fatigue scale recognition under more task conditions and environmental conditions.

## Figures and Tables

**Table 1:** Fatigue self-assessment form.

Fatigue Statement	Never	Sometimes	Regularly	Often	Always
1. I am bothered by fatigue	1	2	3	4	5
2. I get tired very quickly	1	2	3	4	5
3. I don’t do much during the day	1	2	3	4	5
4. I have enough energy for everyday life	1	2	3	4	5
5. Physically, I feel exhausted	1	2	3	4	5
6. I have problems starting things	1	2	3	4	5
7. I have problems thinking clearly	1	2	3	4	5
8. I feel no desire to do anything	1	2	3	4	5
9. Mentally, I feel exhausted	1	2	3	4	5
10. When I am doing something, I can concentrate quite well	1	2	3	4	5

## Data Availability

Some or all data, models, or codes that support the findings of this study are available from the corresponding author upon reasonable request.
